# Tropical Co-infection in a Tertiary Care Center in South India: A Case Report

**DOI:** 10.7759/cureus.67487

**Published:** 2024-08-22

**Authors:** Jibin Simon, Ananthakumar Perumal Kumaresan, Utham Chand, Tirumalasetty Sriharsha, Sharan Bose

**Affiliations:** 1 Internal Medicine, Saveetha Medical College and Hospitals, Saveetha Institute of Medical and Technical Sciences, Saveetha University, Chennai, IND

**Keywords:** co-infection, tropical fever, salmonella paratyphi infection, dengue fever, leptospirosis

## Abstract

Tropical co-infections, characterized by overlapping clinical manifestations and the potential for diagnostic confusion, pose significant challenges in the management of febrile illnesses in endemic regions. This case report presents a 33-year-old male with a five-day history of fever, gastrointestinal symptoms, and dry cough, ultimately diagnosed with co-infections of dengue, leptospirosis, and *Salmonella paratyphi A*. This case underscores the challenges posed by the overlapping clinical features of endemic tropical diseases, emphasizing the necessity for comprehensive diagnostic strategies and tailored treatment protocols in managing febrile illnesses in endemic regions. Clinicians must also consider serological cross-reactivity when interpreting diagnostic tests, as it can complicate the identification of co-infections and impact treatment decisions, necessitating vigilance and an integrated approach in clinical practice.

## Introduction

South India is home to endemic diseases, including dengue, scrub typhus, and leptospirosis, making tropical infections a serious health risk. The overlapping clinical characteristics and the possibility of co-infection make these infections difficult to diagnose and determine which infection is the primary one when an infectious syndrome presents itself [1].

Dengue is caused by the dengue virus, transmitted by *Aedes* mosquitoes. It is characterized by sudden-onset high fever, severe headache, retro-ocular pain, joint and muscle pain, rash, and mild bleeding symptoms. Dengue can progress to severe forms such as dengue hemorrhagic fever (DHF) and dengue shock syndrome (DSS), particularly in cases of secondary infection or in individuals with underlying health conditions [2].

Leptospirosis is caused by *Leptospira *bacteria and is typically transmitted through contact with water contaminated by animal urine. The infection can present as a mild, flu-like illness or progress to more severe forms involving jaundice, renal failure, and bleeding disorders. In tropical regions, leptospirosis often occurs in areas with poor sanitation and during monsoon seasons [3].

*Salmonella typhi* is the primary cause of enteric fever. In recent decades, *Salmonella paratyphi A* has become a significant cause of enteric fever. *Salmonella* often infects humans through contaminated food or water [4]. A clinical observation of *S. paratyphi A* using the paratyphoid human challenge model revealed that *S. paratyphi A*'s pathogenesis is identical to that of *S. typhi* [5]. Paratyphoid fever, like typhoid fever, causes feverish illness and, in severe cases, gastrointestinal bleeding, altered mental status, intestinal perforation, and death [4].

In South India, where these infections are prevalent, distinguishing between them is crucial. The overlapping symptoms of these tropical infections, combined with the potential for co-infection, necessitate a comprehensive diagnostic strategy. Laboratory tests, clinical evaluation, and patient history all play roles in the accurate identification of these diseases. Here, we report the case of a 33-year-old male patient with no known comorbidities who presented with a five-day history of fever, accompanied by gastrointestinal symptoms, including loose stools and vomiting, and an intermittent dry cough. Initial laboratory findings revealed leukopenia, thrombocytopenia, and elevated liver enzymes, prompting a thorough diagnostic evaluation. This case emphasizes the importance of recognizing and treating febrile diseases in areas where tropical infections are common. To improve patient outcomes and manage the complications related to these endemic diseases, thorough screening and suitable treatment options are essential.

## Case presentation

A 33-year-old male patient with no known comorbidities presented with a five-day history of fever, not associated with chills or rigors. He reported four to five episodes of loose stools over one day, which were non-bloody and non-foul-smelling, and three episodes of non-bloody, non-bilious vomiting three days prior. Additionally, he experienced an intermittent dry cough but denied any breathlessness, chest pain, dysuria, abdominal pain, throat pain, or headache.

On a general physical examination, his vital signs revealed a pulse rate of 112 beats per minute and a blood pressure of 90/60 mmHg. Oxygen saturation in room air was 97%, and the respiratory rate was 24 breaths per minute. The patient exhibited no signs of pallor, icterus, cyanosis, clubbing, pedal edema, or lymphadenopathy.

On systemic examination, the cardiovascular system (CVS) revealed normal heart sounds (S1, S2). The respiratory system (RS) had normal vesicular breath sounds. The abdomen was soft and non-tender, and the central nervous system (CNS) showed no focal neurological deficits.

The patient's complete blood count (CBC) revealed a total leukocyte count of 2880 cells/mm^3^, with a differential showing 70% neutrophils and 0% eosinophils. The platelet count was recorded at 0.96 lakh/mm^3^, and hemoglobin (Hb) was 14 g/dL. The peripheral blood smear was normal, with no evidence of parasites for malaria or filaria. Liver function tests (LFT) indicated elevated liver enzymes with aspartate aminotransferase (AST/SGOT) at 184 IU/L and alanine aminotransferase (ALT/SGPT) at 72 IU/L. Serum albumin was low at 3.6 g/dL, and total protein was 7.1 g/dL. Renal function tests showed a serum creatinine level of 0.9 mg/dL and a serum urea level of 24 mg/dL. Serum electrolytes were within normal limits. The erythrocyte sedimentation rate (ESR) was 40 mm/hour, and C-reactive protein (CRP) was significantly elevated at 69 mg/L. Serological testing for hepatitis B surface antigen (HBsAg) and hepatitis C virus (HCV) was non-reactive. Dengue non-structural protein 1 (NS1) antigen was negative, but dengue IgM antibodies were positive.

Serial monitoring of platelet counts demonstrated an initial decline to 80,000 cells/mm^3^, followed by an improvement. The patient was managed conservatively with intravenous (IV) fluids and antipyretics. Due to persistent fever spikes, blood cultures were obtained, and the patient was empirically started on IV ceftriaxone at a dose of 1 g twice daily. Blood cultures subsequently grew *S. paratyphi A*, which was susceptible only to azithromycin, showed intermediate sensitivity to chloramphenicol, ceftriaxone, co-trimoxazole, and ampicillin, and was resistant to tetracycline. Consequently, the treatment regimen was adjusted to include oral azithromycin at 1 g once daily.

Given the rising trend of liver enzymes and persistent fever, additional investigations for scrub typhus and leptospirosis were performed due to their endemicity in the region. Scrub typhus IgM was negative, while leptospirosis IgM returned positive. Considering azithromycin's efficacy in treating leptospirosis, the patient continued this therapy along with ceftriaxone, which was retained based on its intermediate susceptibility profile in the blood culture and sensitivity report. Tables 1, 2 summarize the patient's initial laboratory and microbiological investigations, respectively.

**Table 1:** Laboratory investigations *Values that are outside the normal range and may be significant in the context of dengue, leptospirosis, and paratyphoid co-infection.

Test	Result	Normal Range
Total leukocyte count	2880 cells/mm^3^ *	4000–10000 cells/mm^3^
Neutrophils	70%	40%–70%
Eosinophils	0% *	1%–6%
Platelet count	0.96 lakh/mm^3^ *	1.5–4.5 lakh/mm^3^
Hemoglobin (Hb)	14 g/dL	13.8–17.2 g/dL
Aspartate aminotransferase (AST/SGOT)	184 IU/L *	10–40 IU/L
Alanine aminotransferase (ALT/SGPT)	72 IU/L *	7–56 IU/L
Serum albumin	3.6 g/dL	3.5–5.0 g/dL
Total protein	7.1 g/dL	6.0–8.0 g/dL
Serum creatinine	0.9 mg/dL	0.6–1.2 mg/dL
Serum urea	24 mg/dL	7–20 mg/dL
Erythrocyte sedimentation rate (ESR)	40 mm/hour *	0–15 mm/hour (men), 0–20 mm/hour (women)
C-reactive protein (CRP)	69 mg/L *	0–10 mg/L

**Table 2:** Microbiological investigations

Test	Result	
Blood culture	Salmonella paratyphi A	
Hepatitis B surface antigen (HBsAg)	Non-reactive	
Hepatitis C virus (HCV)	Non-reactive	
Dengue non-structural protein 1 (NS1)	Negative	
Dengue IgM antibodies	Positive	
Scrub IgM antibodies	Negative	
Leptospira IgM antibodies	Positive	

The patient subsequently became afebrile, with improving platelet counts and a downward trend in liver enzyme levels. After being afebrile for more than 48 hours, he was discharged with a prescription for oral azithromycin, 1 g once daily, to complete a seven-day course.

## Discussion

This case report involves a 33-year-old male patient who presented with a five-day history of high-grade intermittent fever, gastrointestinal symptoms, and an intermittent dry cough. Initial investigations revealed significant leukopenia, thrombocytopenia, and elevated liver enzymes. Serological tests confirmed dengue infection, while blood cultures identified *S. paratyphi A*. Despite initial treatment with ceftriaxone, the patient's persistent fever and worsening LFT necessitated further evaluation, which ultimately revealed a leptospirosis co-infection.

In the differential diagnosis of this patient's febrile illness, malaria, typhoid fever, and scrub typhus were considered due to their endemic presence in the region. Malaria was ruled out based on negative blood smear results for *Plasmodium* species. Typhoid fever was differentiated from other forms of salmonellosis by blood culture results identifying *S. paratyphi A*, excluding *S. typhi*. Scrub typhus was also evaluated due to similar clinical presentations and endemicity, but a negative serological test for scrub typhus IgM ruled out this diagnosis.

**Figure 1:**
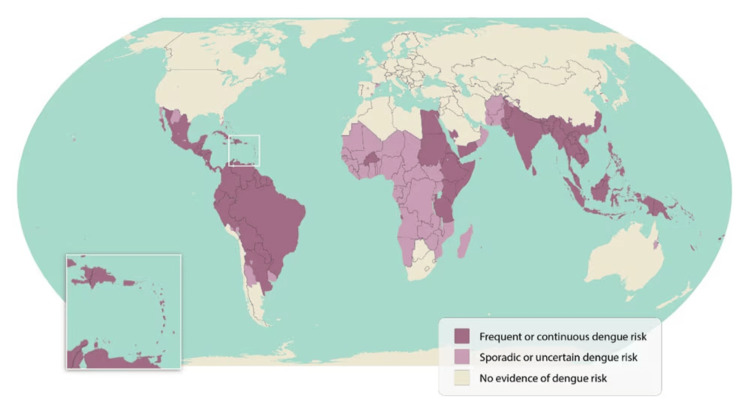
World map highlighting areas of dengue risk Adapted from the Centers for Disease Control and Prevention (June 21, 2024). Areas with a risk of dengue [6].

**Figure 2:**
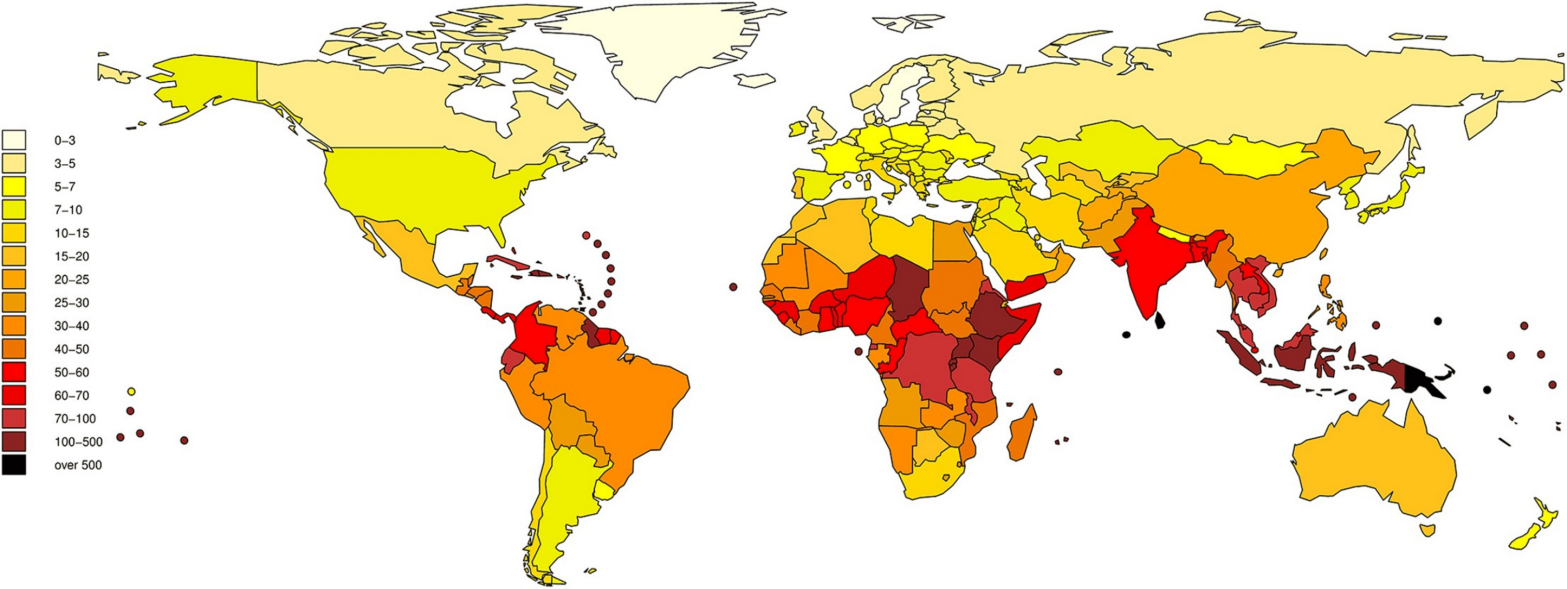
Burden of leptospirosis in terms of DALYs (Disability Adjusted Life Years)/100,000 per year Adapted from Torgerson PR et al. (2015) [7].

The co-infection of leptospirosis and dengue is increasingly recognized, particularly in tropical regions where both pathogens are endemic, as shown in Figures 1, 2 [6,7].

Dengue and leptospirosis share similar clinical presentations, including fever, myalgia, and headache, which can complicate the diagnosis and management [8,9]. Appropriate fluid resuscitation during the critical phase of dengue is the cornerstone of care; there are no definitive curative drugs for dengue [10]. A study reviewing co-infection cases highlighted the challenges in distinguishing between these diseases due to their overlapping symptoms and emphasized the importance of considering both diagnoses in endemic areas [1].

In addition to dengue and leptospirosis, the identification of *S. paratyphi A* in this patient underscores the complexity of co-infections. Enteric fever caused by *Salmonella* species can present with a similar febrile illness, and co-infection with other tropical diseases further complicates the clinical picture. The necessity for broad-spectrum diagnostic approaches and tailored treatment regimens is evident in such cases. Due to widespread resistance to previous first-line antimicrobials, the World Health Organization (WHO) currently recommends empiric treatment with azithromycin, ciprofloxacin, or ceftriaxone for infections caused by *S. typhi* or *paratyphi* [11]. Given that azithromycin is effective against both paratyphoid and leptospirosis infections, patients with a high probability of co-infection could receive treatment with antibiotics that are effective against both illnesses, as was the case in our instance [11,12].

According to Indian recommendations, a positive *Leptospira* IgM serology must be confirmed by microscopic agglutination test (MAT) or culture, which takes time and is only available in reference laboratories, for the diagnosis of leptospirosis. As a result, the MAT for leptospirosis confirmation could not be performed. Consequently, very sensitive and specific tests, such as reverse transcription-polymerase chain reaction (RT-PCR) for dengue and PCR for leptospirosis, are required for a conclusive diagnosis. However, only reference facilities and specialty labs have access to PCR for many infectious illnesses [13]. Therefore, serology is the only laboratory test that is accessible for diagnosis in nations with limited resources. Furthermore, serological cross-reactivity between dengue and leptospirosis antibodies has been documented, which can lead to diagnostic confusion. *Leptospira* and dengue virus antibodies may cross-react in serological tests, complicating the interpretation of results [13]. In countries with limited resources, serology for IgM antibodies may be useful for diagnosing or differentiating between leptospirosis and dengue. To reach a conclusive diagnosis, serological tests that use antibody detection should be interpreted cautiously due to the limitations of cross-reactivity. In endemic locations, specific laboratory tests such as PCR for *Leptospira* and dengue must be carried out for early infection confirmation [13].

## Conclusions

This case report underscores the complexities of managing co-infections in tropical regions, particularly those involving dengue, *S. paratyphi A*, and leptospirosis. The empirical use of azithromycin, which is effective against *Salmonella* and *Leptospira*, resulted in clinical improvement. This case highlights the necessity of considering multiple etiologies in endemic areas, the careful interpretation of serological tests due to potential cross-reactivity, and the importance of adaptable treatment plans that cover multiple pathogens. Comprehensive diagnostic strategies and tailored therapies are essential for managing such complex febrile illnesses effectively.
